# Preparation of Electrode Coke and Determination of Its Physico-Chemical Properties

**DOI:** 10.3390/molecules30234539

**Published:** 2025-11-24

**Authors:** Aigul T. Ordabaeva, Zainulla M. Muldakhmetov, Mazhit G. Meiramov, Sergey V. Kim, Erbolat E. Kuanyshbekov, Shuga B. Kasenova

**Affiliations:** 1 Institute of Organic Synthesis and Chemistry of Coal of Kazakhstan Republic, Alikhanov Str., 1, Karaganda 100012, Kazakhstan; 2 Laboratory of Thermochemical Processes, Zh. Abishev Chemical-Metallurgical Institute, Karaganda 100009, Kazakhstan

**Keywords:** electrode coke, coal tar, pitch, heat capacity, dielectric constant

## Abstract

The physico-chemical and electrophysical properties of carbon coke obtained by coking composite mixtures of pitches isolated from coal tar coking plants of JSC “Shubarkol Komir” and “Qarmet” are investigated. The component composition of the initial resins and the obtained pitches was determined by gas–liquid chromatography methods. The purpose of this study was to identify the patterns of influence of the composition of composite mixtures of pitches isolated from coal tar coking plants of JSC “Shubarkol Komir” and “Qarmet”, as well as heat treatment parameters (temperature 800–1000 °C, duration 4–6 h), on the thermophysical and electrophysical properties of electrode coke, with the determination of optimal conditions for obtaining a material combining low ash content and high carbon content. It was found that the content of phenols and paraffins in the resin of “Shubarkol Komir” is approximately 25% of each component. It is shown that the properties of the final coke depend on the ratio of the mixed pitches (1:1, 1:2, 2:1) and coking conditions (temperature 800–1000 °C, duration 4–6 h). Optimal characteristics (minimal ash content of 0.4%, maximal carbon content of 97.75%) were achieved with a pitch ratio of 1:2 and a temperature of 1000 °C for 6 h. A specific heat capacity in the range of 298–448 K was measured calorimetrically for this sample, where a type II phase transition was detected at 373 K. Electrophysical measurements in the range of 293–483 K revealed a complex temperature dependence of the resistance characteristic of a semiconductor with two sections of a narrow band gap (~0.67 eV and ~0.55 eV). The novelty of the work consists in a comprehensive study of composite mixtures of coal tar pitches and the influence of heat treatment parameters on the formation of thermophysical and electrophysical properties of electrode coke. For the first time, signs of a type II phase transition have been identified for this type of coke material and gigantic permittivity values (up to 10^9^) have been recorded, indicating its potential as a functional carbon material.

## 1. Introduction

Coal tar, a complex multicomponent mixture of aromatic and heterocyclic compounds formed during the coking of bituminous coals, is a valuable secondary raw material for the chemical and carbon industries [1,2,3]. One of its key processing products is coal tar, which is widely used as a binding component in the production of electrodes, graphitized products, and refractory materials [4,5]. The properties of pitch and, as a result, the characteristics of the resulting carbon material are largely determined by the composition of the initial resin and its fractionation conditions and parameters [3,5,6].

With the growing demand for high-quality electrode materials, especially in the aluminum and steel industries, there is a need to develop approaches to managing the properties of pitches. One of the promising directions is the creation of composite mixtures of pitches of various origins, which makes it possible to compensate for the shortcomings of individual components and optimize the characteristics of the final product [7,8,9,10]. Despite well-known work in this field, systematic studies of the effect of the ratio of pitch and temperature–time parameters of coking on the physico-chemical and, especially, electrophysical properties of electrode coke remain insufficiently studied.

Of particular scientific and practical interest are the data obtained indicating that the studied electrode coke exhibits unusual physical properties: a type II phase transition, semiconductor behavior with a narrow band gap, and values of the dielectric constant e ~ 10^9^. These properties, combined, open up prospects for expanding the field of application of baking cokes beyond the traditional electrode production, in particular, as potential functional materials for microelectronics, sensors, and energy intensive devices. For example, as noted in [11,12], pitch-based carbon materials have already been successfully used as electrodes for supercapacitors and sodium batteries, which confirms the trend towards their use in functional devices. The gigantic permittivity observed in carbon systems, as shown in [13], is often caused by interface effects and may be in demand in microelectronics.

The compositioning of low-ash but slightly coked “Shubarkol Komir” pitch with presumably more coked but ashier “Qarmet” pitch will make it possible, due to the complementary properties of the components, to form a carbon matrix with optimized chemical composition and morphology. It is expected that this approach will simultaneously reduce the ash content and increase the carbon content in coke. Moreover, the formation of a heterogeneous nanostructure with developed interphase boundaries can lead to the manifestation of functional electrophysical properties, in particular, narrow-band semiconductor conductivity and a gigantic dielectric constant (ε ~ 10^8^–10^9^), probably due to interface polarization.

The purpose of this work is a systematic study of the effect of the component composition of coal tar (JSC “Shubarkol Komir” and “Qarmet”), the ratio of the pitches obtained from them (1:1, 1:2, 2:1) and coking conditions (temperature 800–1000 °C, duration 4–6 h) for the structural, thermophysical, and electrophysical characteristics of electrode coke. For the first time for such materials, a comprehensive analysis of the temperature dependence of the specific heat capacity was carried out, a phase transition was revealed at 373 K, the parameters of semiconductor conductivity (band gap ~0.67 and ~0.55 eV) were determined, and the values of permittivity reaching 10^9^ were recorded.

Despite significant progress in the development of technologies for producing electrode coke, problems of the instability of the composition of the initial coal tar remain relevant due to differences in raw coal, coking modes, and purification technologies, which lead to fluctuations in the quality of pitch and, as a result, coke [14].

In addition, individual pakes are characterized by insufficient coking capacity, which is due to their physico-chemical parameters. In particular, the pitch of JSC Shubarkol Komir has a low softening temperature (71–75 °C), a high yield of volatile substances (up to 86%), and a low content of the α fraction (7.24%), which may indicate a low degree of condensation and polymerization readiness of hydrocarbon structures [15]. These characteristics reduce the ability of the material to form a dense sintered carbon matrix during heat treatment, which leads to a decrease in strength and a decrease in coke yield.

The high ash content of the pitch from some industries (including Qarmet) limits the use of the resulting coke in the aluminum industry, where the permissible ash content should not exceed 0.5%.

There is a lack of systematic data in the scientific literature on the effect of the composition of pitches with various rheological and thermal properties on the complex of physico-chemical and, especially, electrophysical characteristics of the final coke, despite the growing interest in its functional use.

Thus, the task arises of purposefully managing the properties of coke by compensating for the disadvantages of one component at the expense of the advantages of the other—for example, the low ash content of the Shubarkol pitch and the high coking capacity of the Qarmet pitch.

## 2. Results

### Determination of the Physico-Chemical Properties of the Feedstock (Coal Tar Coking Chemical Plants JSC “Shubarkol Komir” and “Qarmet”)

**Figure 1:**
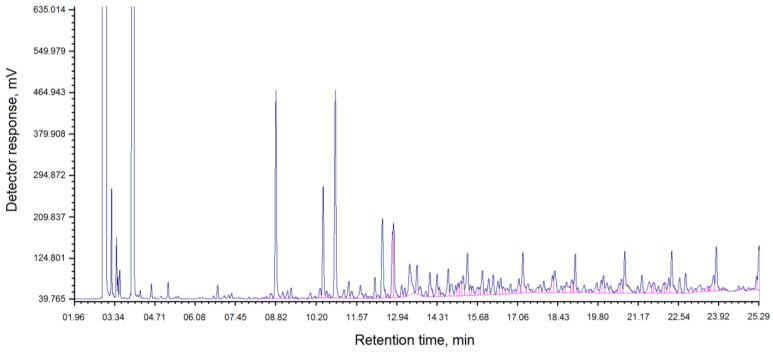
Chromatogram of the component composition of the resin “Shubarkol Komir”.

Table 1 and Figure 1 show the component composition of coal tar “Shubarkol Komir”.

The chromatogram of the component composition of the resin “Shubarkol Komir” is shown in Figure 1.

As can be seen from Table 1 and the chromatogram in Figure 1, the content of phenol and its derivatives is 24.96% and paraffins are 24.96%.

Table 2 shows the main physico-chemical characteristics of pitch obtained from the coal tar of JSC Shubarkol Komir.

The composition of fractions of pitch “Shubarkol Komir”, soluble in toluene, is shown in Table 3.

**Figure 2:**
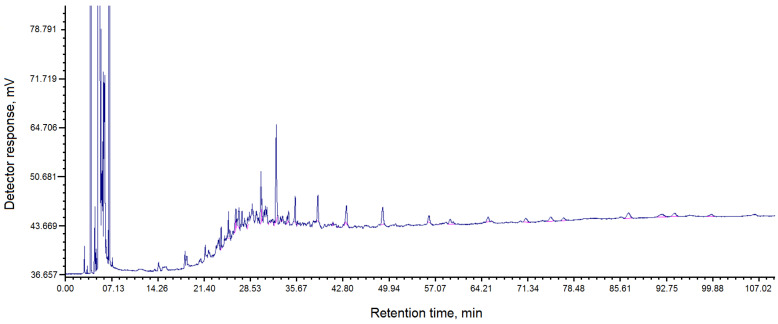
Chromatogram of the composition of fractions of pitch “Shubarkol Komir”, soluble in toluene.

The chromatogram of the composition of fractions of pitch “Shubarkol Komir”, soluble in toluene, is shown in Figure 2.

The composition of fractions of pitch “Shubarkol Komir”, soluble in quinoline, is presented in Table 4.

**Figure 3:**
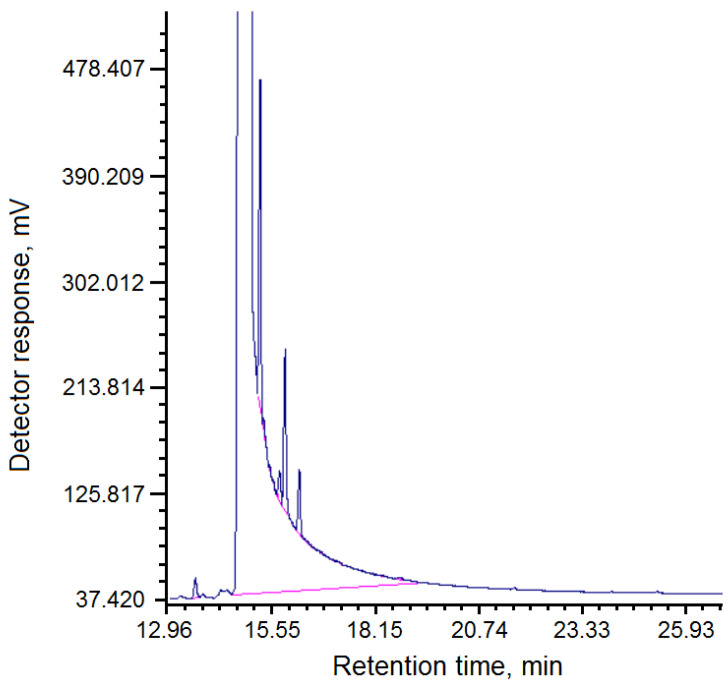
Chromatogram of the composition of fractions of pitch “Shubarkol Komir”, soluble in quinoline.

The chromatogram of the composition of fractions of pitch “Shubarkol Komir”, soluble in quinoline, is shown in Figure 3.

As can be seen from the results of studying the physico-chemical properties of the feedstock, the resulting pitch belongs to low-temperature (soft) pitches. Such pitches contain a large number of highly volatile and low-molecular-weight components, which reduce the temperature of transition to the plastic state [4,16].

As a result of the coking of the obtained composite mixtures under various conditions, samples of electrode coke were obtained, differing in ash content and contents of C, H, and S. The results of the coking of the obtained composite mixtures are presented in Table 5.

An increase in the coking temperature from 800 to 1000 °C leads to a monotonous decrease in ash content by 70% and an increase in carbon content by ~1.7% (Table 5), which may indicate increased dehydrogenation, the removal of volatile components, and an increase in the degree of aromatization of the carbon matrix. The decrease in ash content is probably due to the partial removal of mineral impurities in the form of volatile compounds or their sintering and incorporation into the carbon matrix, which reduces their share in the total mass of coke. The most pronounced effect was achieved with a pitch ratio of 1:2, which indicates the complementary effect of low-ash pitch “Shubarkol Komir” and, presumably, more coked pitch “Qarmet”.

The data in Table 5 demonstrate a clear dependence of the elemental composition of coke on the heat treatment conditions: with an increase in temperature from 800 to 1000 °C and an increase in the holding time from 4 to 6 h, a monotonous increase in carbon content (from ~95% to 97.75%) and a decrease in concentrations of hydrogen and sulfur (H: from ~1.0% to 0.51%; S: from ~0.8% to 0.47%). These changes indicate an intensive thermal degradation of the initial baking matrix and the subsequent structural restructuring of the carbon deposit.

A decrease in the hydrogen content indicates the dehydrogenation and condensation of aromatic nuclei, accompanied by the loss of aliphatic and heteroatomic substituents, a process typical of the high-temperature coking of coal pitches [4,5]. A simultaneous decrease in sulfur (by ~44%) is possible due to the thermal decomposition of organic sulfur compounds (thiophenes, mercaptans, etc.) with the release of volatile products such as HS and carbon disulfide, which is consistent with data from thermogravimetric and mass spectrometric studies of pitches [14,15].

The purest and carbonized coke (C = 97.75%, ash content = 0.40%) was obtained at a pitch ratio of 1:2 at 1000 °C and 6 h, which confirms the effectiveness of the compositional approach: due to the complementary properties of the components, not only a reduction in mineral impurities is achieved, but also the formation of a more ordered aromatic structure. Such a material is characterized by signs of partial graphitization, which opens up the possibility of its use, not only as a structural (electrode), but also as a functional carbon material.

This sample was selected for an in-depth study of thermophysical and electrophysical properties. It has been established that the temperature dependence of the specific heat capacity shows a pronounced anomaly at 373 K—a sharp peak not accompanied by latent heat, which corresponds to a type II phase transition.

Table 6 shows the experimental values of the specific heat capacity of the Shubarkul–Qarmet electrode coke sample (1:2).

It can be seen from the experimental data that a non-monotonic increase in heat capacity is observed, and at a temperature of 373 K there is a type II phase transition.

It should be noted that an anomaly in the form of a peak on the curve of the temperature dependence of the specific heat at 373 K may indicate the presence of a phase or relaxation transition. Interestingly, in the same temperature range, there is a change in the nature of the dependence of electrical resistance on temperature: in the range of 293–373 K, resistance decreases with increasing temperature (behavior typical for semiconductors), whereas, in the range of 373–393 K, there is a short-term increase in resistance. This coincidence indicates a possible connection between the thermodynamic anomaly and the rearrangement of the electron structure or interfacial boundaries in the material. However, additional studies, including measurements of charge carrier density and mobility, are required to unambiguously interpret the nature of the transition.

Taking into account the temperature of the phase transition, the equations of the temperature dependence of the specific heat capacity [J/(g * K)] are derived:

(1) $$C_{p}=(2.53\pm 0.09)-(0.837\pm 0.03)\cdot 10^{-3}T-(1.35\pm 0.05)\cdot 10^{-5}T^{-2}\cdot (298-373\cdot K)$$

(2) $$C_{p}=(4.24\pm 0.16)-(8.04\pm 0.3)\cdot 10^{-3}T\cdot (298-373\cdot K)$$

(3) $$C_{p}=-\left(0.22\pm 0.01\right)+\left(3.17\pm 0.12\right)\cdot 10^{-3}T\cdot (298-373\cdot K)$$

For the temperature ranges under consideration, the value of the average random error was used to determine the coefficient error in the dependence equations $C_{p}^{0}$ ~ *f*(T).

The results of the electrophysical measurements of the obtained electrode coke material in the temperature range of 293–483 K and at an alternating electrical signal frequency of 1 kHz are presented in Table 7.

**Figure 4:**
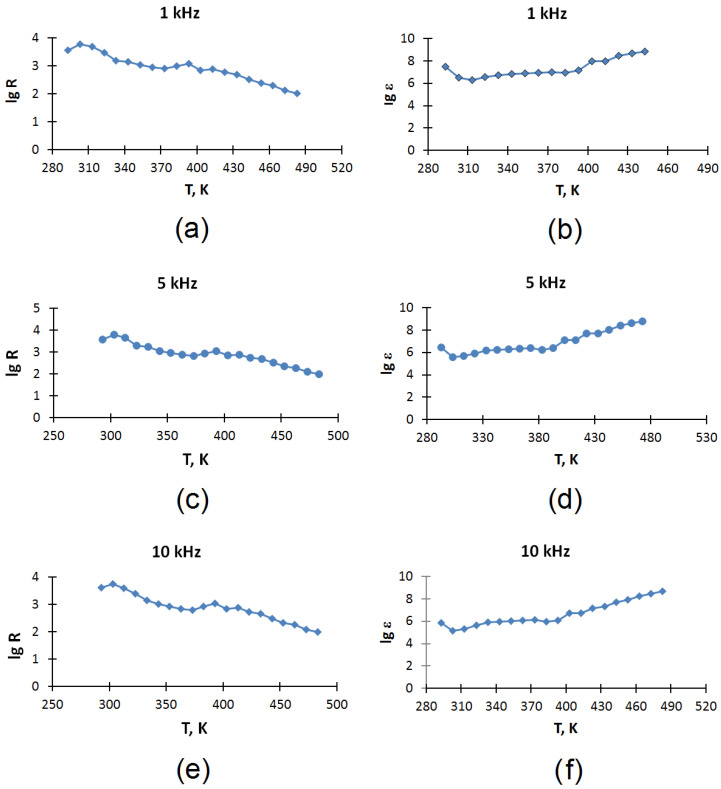
(**a**–**f**) Temperature dependences of the dielectric constant (ε) and electrical resistance (R) of an electrode coke sample from a Shubarkol–Qarmet mixture in the temperature range of 293–483 K at frequencies of 1, 5, 10 kHz: (**a**) Temperature dependence of electrical resistance (lg R) at 1 kHz; (**b**) Temperature dependence of dielectric permittivity (lg ε) at 1 kHz; (**c**) Temperature dependence of electrical resistance (lg R) at 5 kHz; (**d**) Temperature dependence of dielectric permittivity (lg ε) at 5 kHz; (**e**) Temperature dependence of electrical resistance (lg R) at 10 kHz; (**f**) Temperature dependence of dielectric permittivity (lg ε) at 10 kHz.

Table 7, Table 8 and Table 9 also indicate the relative measurement errors of electrical capacity δ_C_ and electrical resistance δ_R_ obtained as a result of three parallel measurements (n = 3). The values of the dielectric constant (ε) shown in Figure 4, as well as in Table 7, Table 8 and Table 9, are the result of calculation using formula (10), which uses the average values of three parallel measurements (n = 3) of electrical capacity (C). The average values of electrical resistance (R) are also obtained as a result of three parallel measurements (n = 3).

The results of the electrophysical characteristics of the obtained electrode coke in the temperature range of 293–483 K and at an alternating electrical signal frequency of 5 kHz are presented in Table 8.

The results of the measurements of the electrophysical characteristics of the obtained electrode coke in the temperature range of 293–483 K and at an alternating electrical signal frequency of 10 kHz are presented in Table 9.

The temperature dependences of the dielectric constant (ε) and electrical resistance (R) of an electrode coke sample from a Shubarkol–Qarmet mixture in the temperature range of 293–483 K at frequencies of 1, 5, and 10 kHz are shown in Figure 4.

Analysis of the temperature dependence of conductivity and dielectric properties.

It can be seen from the experimental data that the material (electrode coke obtained from a mixture of Shubarkol and Qarmet pitches in a ratio of 1:2) demonstrates temperature-dependent conductivity. According to the nature of the change in resistance with temperature, three sections can be distinguished:

- 293–373 K—a decrease in resistance is observed with increasing temperature, which is typical for the semiconductor type of conductivity;
- 373–393 K—resistance increases, which corresponds to metallic behavior;
- 393–483 K—the resistance decreases again with increasing temperature, which indicates a return to semiconductor behavior.

Calculation of the band gap (ΔE) in the range of 293–373 K. Readings at 293 *K*—3.56 *lg R*, 373 *K*—2.90 *lg R:*

(4) $$\Delta E=\frac{2\;\times \;0.000086173\;\times \;293\;\times \;373}{0.43\;(373\;-\;293)}\mathrm{lg}\frac{3.56}{2.90}=0.67eB$$

In this range, the band gap is 0.67 eV and can be attributed to narrow-probe semiconductors.

Calculation of the band gap (ΔE) in the range of 393–483 K. The reading at 393 K is 3.09 *lg R*, 483 K is 2.01 *lg R*:

(5) $$\Delta E=\frac{2\;\times \;0.000086173\;\times \;393\;\times \;483}{0.43\;(483-393)}\mathrm{lg}\frac{3.09}{2.01}=0.55eB$$

In this range, the band gap is 0.55 eV and can be attributed to narrow-probe semiconductors.

The material (electrode coke obtained from a mixture of Shubarkol and Qarmet pitches in a ratio of 1:2) also has very high values of dielectric constant (ε).

In addition to the electrical characteristics, the material exhibits very high values of dielectric constant (ε). Values of ε reach the following levels:

More than 107 at low temperatures (293–373 K);

108–109 when the temperature rises above 393 K.

Ferroelectrics or materials with a gigantic permittivity value will be stunned by similar values.

## 3. Discussion

The optimization of the mixture composition and coking mode made it possible to overcome the technological limitations associated with the use of Shubarkol Komir pitch, which has unfavorable characteristics: low softening temperature (71–75 °C), high yield of volatile substances (86%), and low content of the coke base (α-fraction—7.24%). Despite these disadvantages, Shubarkol Komir pitch has an exceptionally low ash content (0.16%), which makes it a valuable component in the composition. In combination with Qarmet pitch, which presumably has a higher coking capacity, it was possible to achieve optimal coke characteristics: minimum ash content—0.4% and maximum carbon content—97.75% (with a pitch ratio of 1:2, a temperature of 1000 °C, and a duration of 6 h).

Thus, the low ash content of the Shubarkol Komir pitch compensates for its low coking capacity, and the high coking capacity of the Qarmet pitch ensures the formation of a dense, graphitizing structure with a high yield of carbon residue. This approach is fully consistent with current trends in the coal industry, where pitch mixing is used to optimize rheological and coking properties [4].

The obtained optimal sample became the basis for further fundamental studies of its physical properties, which revealed its potential, not only as a structural material, but also as a functional material.

For the first time, a type II phase transition at 373 K (100 °C) was recorded for baking coke, which was confirmed both calorimetrically (peak heat capacity, Table 6) and electrophysically (change in the type of conductivity from semiconductor to metallic and vice versa, Table 7, Table 8 and Table 9), which may indicate a deep restructuring of the electronic structure of the material.

The observed permittivity values go beyond the limits of conventional dielectrics and are closer to the effects described by the Maxwell–Wagner–Sillars model of interface polarization. In heterogeneous carbon materials containing regions with different conductivities (for example, graphite-like domains and amorphous inclusions), charges accumulate at the phase boundaries when an alternating electric field is applied [17,18]. This leads to a significant increase in the effective dielectric constant, especially in the low-frequency range and with increasing temperature, when the mobility of charges increases.

The compositing of low-ash but slightly coked Shubarkol Komir pitch with presumably more coked Qarmet pitch can contribute to the formation of a carbon matrix with optimized morphology and chemical composition. 

This approach can simultaneously reduce the ash content and increase the fixed carbon content in the final coke. The formation of structural heterogeneity with interfacial boundaries differing in the level of conductivity and polarizability can potentially improve the electrophysical characteristics of the material, including partial semiconductor conductivity and increased dielectric constant.

Gigantic dielectric constant (ε ~ 108–109 at T > 393 K) is characteristic of materials with strong interface polarization [14]. This property opens up prospects for the use of coke, not only as an electrode, but also in microelectronics, sensors, and energy-intensive devices.

Possibly, in parallel with the changes in the structure, the mechanisms of interphase and dipole polarization are enhanced, which leads to an increase in the dielectric constant ε in a certain temperature-frequency range.

This mechanism is most likely for heterogeneous carbon materials containing phases with significantly different conductivity—in our case, these are graphite-like more conductive regions and amorphous carbon-less conductive fragments of the coke matrix. When an alternating electric field is applied, mobile charge carriers (electrons or holes) are able to migrate inside conductive regions, but their passage through the boundaries with less conductive areas is limited. This leads to the accumulation of charge at the interfacial boundaries, the formation of an effective interfacial dipole response, and, as a result, a significant increase in the measured dielectric constant.

The characteristics observed in this work correspond to the features of MWS polarization described in the literature for other heterogeneous systems. First, a pronounced frequency dispersion was revealed—the values of ε decrease by 1–2 orders of magnitude with an increase in frequency from 1 to 10 kHz (Table 7, Table 8 and Table 9), which is consistent with the limited rate of charge accumulation at the phase boundaries at high frequencies. Secondly, a noticeable increase in ε was recorded at temperatures above 393 K, simultaneously with a change in the nature of the resistance dependence, which may indicate the thermally activated mobility of charge carriers and increased interphase polarization.

## 4. Materials and Methods

### 4.1. Determination of the Physico-Chemical Properties of the Feedstock (Coal Tar Coking Chemical Plants JSC “Shubarkol Komir” and “Qarmet”)

The analysis of the component composition of coal tar from the coke chemical production of JSC Shubarkol Komir was carried out using gas–liquid chromatography (GLC). The Crystalllux 4000 M chromatograph (NPF “Meta-chrome”, Yoshkar-Ola city, Russia), equipped with a PID/PID detector module and a ZB-5 cone measuring 30 m × 0.32 mm × 0.25 microns, was used for analysis. The chromatograms were processed using the NetChrom v2.1 program. The component composition of Shubarkol Komir coal tar is shown in Table 1, and the chromatogram is shown in Figure 1.

To remove water from coal tar, JSC Shubarkol Komir used an IR 1-LT rotary evaporator (LABTEX, Moscow city, Russia). To do this, 100 g of the initial resin was placed in a 250 mL round-bottomed flask and evaporated on a rotary evaporator at a water bath temperature of 50 °C and a pressure of 20 mmHg until the water was completely removed. After evaporation, the resin weight was 92 g.

Further, three fractions were isolated using vacuum distillation of resin from JSC Shubarkol Komir:

The 1st fraction was obtained at a boiling point of up to 150 °C and a pressure of 10 mmHg (17 g);

The 2nd fraction was obtained at a boiling point of 170–190 °C and a pressure of 10 mmHg (23 g);

The 3rd fraction was obtained at a boiling point of 220–250 °C with a pressure of 10 mmHg (4 g).

The mass of the pitch remaining after distillation was 48 g.

The yield of the resin fractions of JSC “Shubarkol Komir” is shown in Table 10.

To assess the applicability of the obtained pitch in electrode production, the main characteristics determining its quality were established. The following parameters were determined: softening temperature, solubility in toluene and quinoline, ash content, and the yield of leucic substances.

The softening temperature of the pitches was determined according to GOST 9950-2020 [19]. The content of substances insoluble in toluene (α fractions) in baking was determined in accordance with GOST 7847-2020 [20]. According to GOST 10200-2017 [21], the mass fraction of substances insoluble in quinoline (α1 fractions) was determined. The yield of volatile substances was determined according to GOST 70547-2022 [22]. The pitch ash content was determined in accordance with GOST 70542-2022 [23].

To determine the softening temperature, crushed dry pitch obtained from coal tar of JSC Shubarkol Komir, weighing 1.6 g, was pressed into a mold with a diameter of 20 mm with a force of 1600 kgf, with a holding time at the final force of 15 s. The tablet was then placed in a device for determining the softening temperature, with an initial glycerol temperature of 40 °C. Next, the temperature was raised at a rate of 5 °C/min. The set softening temperature was 71–75 °C. Using the GRC analysis method, the composition of fractions in the resulting pitch, soluble in toluene and quinoline, was determined (Table 3 and Table 4, Figure 2 and Figure 3).

### 4.2. Preparation of Baking Composites

The analysis of the component composition of pitch obtained from coal tar from the coke chemical production of JSC Shubarkol Komir was also carried out using gas–liquid chromatography (GLC). A Crystallux 4000 M chromatograph was used for the analysis. The table of the component composition of pitch is presented in Table 3, and the chromatogram is shown in Figure 3.

For the preparation of baking composites, pitches obtained by vacuum distillation of coal tar from the coke chemical production of Shubarkol Komir and Qarmet were mixed with each other in the following ratios: 1:1; 1:2; and 2:1.

The mixing of the pitches was carried out in order to optimize their properties to obtain electrode coke with specified characteristics. The mixing of pitches makes it possible to use Shubarkol Komir pitch in areas where pure pitch is not suitable due to its characteristics.

The analysis of the component composition of Qarmet coal tar and pitch obtained as a result of its vacuum distillation was previously presented by the authors in [24].

### 4.3. Production of Electrode Coke

As a result of mixing the pitches obtained by vacuum distillation of coal tar from the coking plants Shubarkol Komir and Qarmet, a composite material was obtained, which was placed in a coking unit. Coking was carried out in the temperature range of 800–1000 °C, and the duration of the process was 4–6 h. The effect of the production conditions on the ash content and the content of C, H, and S is shown in Table 5.

The choice of temperature range (800–1000 °C) and duration (4–6 h) is due to the need to ensure the complete removal of volatile components and the formation of a dense carbon matrix without cracking. According to [4,5], temperatures below 800 °C lead to incomplete coking, while above 1000 °C leads to excessive shrinkage and the loss of the mechanical integrity of the samples.

The determination of the mass fraction of carbon (C) and hydrogen (H) in the resulting coke was carried out according to GOST 2408.1-95 (ISO 625-96) [25]. The mass fraction of nitrogen (N) in the resulting coke was determined according to GOST 28743-93 [26]. The determination of the mass fraction of sulfur (S) was carried out according to the GOST 8606-2015 (ISO 334.2013) methodology [27].

### 4.4. Determination of the Heat Capacity by the Calorimetric Method

The temperature dependence of the heat capacity of a sample of electrode coke obtained from the pitches of the Shubarkol Komir and Qarmet coke chemical plants was measured on a serial IT-S-400 calorimeter (Instrument-making Plant, Aktobe city, Kazakhstan) in the temperature range of 298.15–448 K.

The experiments were carried out in a monotonous, close to linear heating of the sample at an average rate of about 0.1 K per second with temperature differences between the sample and the medium at 3–30 K. With such temperature differences, the time of temperature delay on the heat meter was measured. In one experiment, the temperature dependence of the studied parameter was determined. The measuring circuit of the device provided a measurement of the temperature level from 100 to 400 °C at fixed points after 25 °C using a DC potentiometer and a switch built into the device. The heat meter was a heat flux converter that provided flow measurements, equalized the surface temperature of the sample, and made it possible to calibrate directly in the heat block to account for errors. The duration of measurements in the entire temperature range with the processing of experimental data was no more than 2.5 h. The limit of permissible error of the device according to the passport data was ±10.0%. The principle of operation of the device was based on the comparative method of a dynamic c-calorimeter with a heat meter. The test sample was placed in a metal ampoule of a measuring cell and heated continuously by a heat flow through a heat meter. Every 25 °C of heating, a time delay in the pool temperature relative to the base temperature was measured using a microvoltammeter and a serial stopwatch. The calibration of the device was carried out based on the determination of the thermal conductivity of the CT heat meter. For this purpose, five experiments were conducted with an empty ampoule and the same number with a copper sample.

The thermal conductivity of the heat meter was determined by the following formula:

(6) $$K_{T}=\frac{C_{Cu\;sample}}{\left(\bar{\tau_{TM}}-\bar{{\bar{\tau }}_{T}^{{}^{\circ}}}\right)}$$

where:

$C_{Cu\;sample}$ is the total heat capacity of the copper sample, J/(mol·K);

$\bar{\tau_{TM}}$ is the average value of the delay time on the heat meter in experiments with a copper sample, with

${\bar{\tau }}_{T}^{{}^{\circ}}$—as the average delay time in experiments with an empty ampoule, s.

The total heat capacity of the copper sample was calculated using the following formula:

(7) $$C_{Cu\;sample}=C_{M}\cdot m_{Cu\;sample}$$

where *C_M_* is the tabular value of the specific heat capacity of copper, J/(mol·K); *m_Cu sample_* is the mass of the copper sample, kg.

The value of the specific heat capacity of the test substance was calculated by the following formula:

(8) $$C=\frac{K_{T}}{m_{0}(\tau_{T}\;-\;\tau_{T}^{{}^{\circ}})}$$

where *C_i_* is the specific heat capacity of the test substance;

*K_T_* is the thermal conductivity of the heat meter;

*m*_0_ is the mass of the test substance;

T is the delay time of the temperature on the heat meter;

$\tau_{T}^{{}^{\circ}}$ is the delay time of temperature on the heat meter in experiments with an empty ampoule, s.

Five parallel experiments were conducted at each temperature, the results of which were averaged and processed using mathematical statistics methods.

At each temperature, the standard deviation ($\bar{\delta }$) was estimated for the averaged values of the specific heat according to the following method [28]:

(9) $$\bar{\delta }=\sqrt{\frac{\sum_{i=1}^{n}{\left(C_{i}\;-\bar{\;C}\right)}^{2}}{n\;-\;1}}$$

where *n* is the number of experiments;

*C_i_* is the measured value of the specific heat;

*C* is the arithmetic mean of the measured values of the specific heat.

The operation of the calorimeter was checked by determining the standard heat capacity of α-Al_2_O_3_. Its experimental value [76.0 J/(mol·K)] is in satisfactory agreement with the reference data [79.0 J/(mol·K)] within ~4.0% [29].

To determine the error of the coefficients in the dependence equations $C_{p}^{{}^{\circ}}$ ~ ƒ(*T*), the values of the standard deviation for the temperature ranges under consideration were used.

The temperature dependence of the heat capacity of the electrode coke sample was studied according to the procedure described above. At each temperature (after 25 K), five parallel experiments were performed and their results were averaged by determining the standard deviation ($\bar{\delta }$) for the specific heat.

Experimental values of the specific heat capacity of a sample of coke electrode Shubarkol–Qarmet (1:2) [$C_{p\pm }\bar{\delta }$, J/(g × K)] are shown in Table 6.

### 4.5. Determination of the Electrophysical Characteristics of the Obtained Electrode Coke

Measurements of electrophysical properties were carried out according to the methods in [30,31].

The study of electrophysical properties (dielectric constant and electrical resistance) was carried out by measuring the electrical capacity of samples on a serial device LCR-800 (Good Will Instrument Co., Ltd., Taipei city, Taiwan) at an operating frequency of 1kHz continuously in dry air in a thermostatic mode with a holding time at each fixed temperature.

Plane-parallel samples in the form of disks with a diameter of 10 mm and a thickness of 5–6 mm with a binder additive (~1.5%) were preliminarily produced. The pressing was carried out at a pressure of 20 kg/cm^2^. The resulting disks were fired in a laboratory furnace (SNOL, Utena, Lithuania) at 400 °C for 6 h. Then they were carefully sanded on both sides.

The dielectric constant was determined from the electrical capacity of the sample at known values of the sample thickness and the surface area of the electrodes. The Sawyer–Tower scheme was used to obtain the relationship between electric induction D and electric field strength E. Visual observation of the D (E hysteresis loop) was carried out on a C1-83 oscilloscope (Zolochiv Radio Factory, Zolochiv city, Ukraine) with a voltage divider consisting of a resistance of 6 mOhm and 700 kOhm and a reference capacitor of 0.15 UF. The frequency of the generator was 300 Hz. In all temperature studies, the samples were placed in an oven, and the temperature was measured with a chromel–alumel thermocouple connected to a voltmeter B2-34 (JSC Taganrog Priboy Plant, Taganrog city, Russia) with an error of 0.1 mV. The rate of temperature change was 5 K/min. The value of the dielectric constant at each temperature was determined by the following formula:

(10) $$\varepsilon =\frac{C}{C_{0}}$$

where $C_{0}=\frac{\varepsilon_{0}S}{d}$ is the capacitance of the capacitor without the test substance (air).

The calculation of the band gap (Δ*E*) of the test substance was determined by the following formula:

(11) $$∆E=\frac{2kT_{1}T_{2}}{0.43(T_{2}\;-{\;T}_{1})}$$

where *k* is the Boltzmann constant equal to 8.6173303 10^−5^ eV K^−1^, R_1_ is the resistance at *T*_1_, and *R*_2_ is the resistance at *T*_2_.

Table 1 and Figure 1 below show the results of the electrophysical measurements of the coke material in the range of 293–483 K and at frequencies equal to 1, 5, and 10 kHz.

The dependences of the electrical resistance (*R*), electrical capacity (*C*), and dielectric constant (ε) of the obtained electrode coke on temperature (*T*) at frequencies of an alternating electrical signal of 1, 5, 10 kHz are presented in Table 7, Table 8 and Table 9. Diagrams of temperature dependences are presented in Figure 4.

## 5. Conclusions

This study systematically investigates the influence of the chemical composition of coal tar pitches (from Shubarkol Komir JSC and Qarmet), the blending ratios of the pitches derived from them (1:1, 1:2, and 2:1), and coking conditions (temperature 800–1000 °C, duration 4–6 h) on the physicochemical and electrophysical properties of electrode coke.

It has been established that the compositioning of low-ash but slightly coked “Shubarkol Komir” pitch with, presumably, more coked “Qarmet” pitch makes it possible to effectively compensate for the disadvantages of each component. The optimal coke characteristics—minimum ash content (0.40%) and maximum carbon content (97.75%)—were achieved with a pitch ratio of 1:2 and coking conditions of 1000 °C/6 h, which confirms the expediency of the composite approach in the production of high-quality carbon materials.

For the obtained sample, a type II phase transition was detected for the first time at 373 K, confirmed calorimetrically (peak heat capacity), electrophysically (change in the nature of the dependence of resistance on temperature), and by semiconductor conductivity with two narrow band gap regions (~0.67 eV and ~0.55 eV).

It was also found that the resulting coke has a very large dielectric constant (ε ~ 10^8^–10^9^ at T > 393 K), which exhibits a frequency and temperature dispersion characteristic of interface polarization in heterogeneous carbon systems.

It is important to emphasize that these properties do not allow us to directly assert the practical applicability of coke in microelectronics or sensors without additional tests (DC stability, compatibility with lithographic processes, cyclability, etc.). Nevertheless, the results obtained indicate the potential of this material as an object of fundamental research in the field of functional carbon materials. Similar physical effects have previously been observed in nanostructured coals, reduced graphene oxide, and baking cokes, which opens up prospects for further study of the relationship between composition, structure, and functional properties.

Thus, this work demonstrates the possibility of purposefully controlling the properties of coke by selecting the composition of the initial pitches and heat treatment parameters, which can be used both to produce high-quality electrodes and to develop new functional carbon materials with specified physical characteristics.

## Figures and Tables

**Table 1:** Component composition of coal tar “Shubarkol Komir”.

№	Component	Time, min	Concentration, %
1	Phenol	8.83	5.77
2	Decane	9.35	0.27
3	2-Methylphenol	10.44	3.05
4	3-Methylphenol	10.86	6.9
5	Undecane	11.41	0.31
6	2,6-Dimethylphenol	11.71	0.66
7	2-Ethylphenol	12.20	0.60
8	2,5-Dimethylphenol	12.45	3.12
9	2,4-Dimethylphenol	12.54	0.04
10	3,4-Dimethylphenol	12.80	1.79
11	3-Ethylphenol	12.84	2.49
12	Dodecane	13.63	0.93
13	2-Propylphenol	13.94	0.21
14	Tridecane	15.35	1.88
15	2-tert-Butyl-6-methylphenol	15.76	0.24
16	Tetradecane	17.41	0.50
17	Pentadecane	19.02	1.26
18	Hexadecane	20.70	2.02
19	Heptadecane	22.30	1.73
20	Octadecane	23.82	1.57
21	Nonadecane	25.27	1.76
22	Eicosane	26.78	2.01
23	Heneicosane	30.40	1.87
24	Docosane	32.71	2.08
25	Tricosane	35.52	2.13
26	Tetracosane	39.04	1.82
27	Pentacosane	43.45	1.31
28	Hexacosane	49.06	0.81
29	Heptacosane	56.12	0.44
30	Octacosane	65.19	0.36
31	n-Hexane	65.87	0.38
32	Benzo[a]pyrene	77.22	0.04
33	Triacontane	92.57	0.26
34	Hentriacontane	111.25	0.27
35	Dotriacontane	128.92	0.04
36	Tritriacontane		0.05
	**Identified:**		**51.16**
	**Group**		
	Paraffins	25.77	
	Phenols	24.96	
	**Total**	**50.73**	

**Table 2:** Physico-chemical parameters of pitch obtained from the coal tar of JSC “Shubarkol Komir”.

Indicator	Obtained Pitch
Mass fraction of substances insoluble in quinoline, %, not more than	2.67
Mass fraction of substances insoluble in toluene, %	7.24
Softening temperature, °C, not more than	71–75 °C
Volatile matter yield, %	86
Ash content, %, not more than	0.16

**Table 3:** Composition of fractions of pitch “Shubarkol Komir”, soluble in toluene.

№	Component	Retention Time, min	Concentration, %
1	Octadecane	24.11	2.16
2	Nonadecane	25.23	2.57
3	Eicosane	26.90	4.24
4	Heneicosane	28.19	1.48
5	Docosane	30.31	2.83
6	Tricosane	32.55	18.36
7	Tetracosane	35.85	0.091
8	Pentacosane	38.98	5.45
9	Hexacosane	43.38	5.08
10	Heptacosane	48.97	6.69
11	Octacosane	56.10	2.40
12	Nonacosane	65.47	2.27
13	Triacontane	77.08	1.70

**Table 4:** Composition of fractions of pitch “Shubarkol Komir”, soluble in quinoline.

№	Component	Time, min	Concentration, %
1	Dodecanese	13.70	0.046
2	Quinoline	15.04	98.79
3	Tridecan	15.35	0.016
4	2-tert-Butyl-6methylphenol	15.75	0.066
5	Tetradecane	17.39	0.00049
6	Pentadecane	19.01	0.00078
**Total**			**98.92**

**Table 5:** Effect of production conditions on ash content and content of C, H, S.

Ratio	T, °C	4 h				5 h				6 h			
		Ash, %	C, %	H, %	S, %	Ash, %	C, %	H, %	S, %	Ash, %	C, %	H, %	S, %
Shubarkol + Qarmet (1:1)	800	1.15	95.11	0.97	0.67	1.11	95.14	0.92	0.59	1.01	95.51	0.83	0.57
	900	0.76	95.14	0.88	0.63	0.49	95.34	0.80	0.57	0.41	95.71	0.75	0.49
	1000	0.54	95.34	0.86	0.51	0.50	95.69	0.73	0.47	0.37	95.73	0.70	0.46
Shubarkol + Qarmet (1:2)	800	1.28	96.12	1.05	0.59	1.17	96.45	1.00	0.59	0.77	96.45	0.94	0.55
	900	1.01	96.51	0.98	0.59	0.55	96.73	0.81	0.55	0.41	96.93	0.76	0.51
	1000	0.74	96.81	0.91	0.49	0.48	97.56	0.52	0.49	0.4	97.75	0.51	0.47
Shubarkol + Qarmet (2:1)	800	1.04	94.89	0.99	0.84	0.96	95.09	0.99	0.74	0.76	95.11	0.97	0.68
	900	0.41	95.03	0.88	0.60	0.40	95.24	0.86	0.52	0.34	96.77	0.78	0.47
	1000	0.27	95.92	0.81	0.47	0.26	96.88	0.64	0.45	0.26	97.37	0.51	0.45

**Table 6:** Experimental values of the specific heat capacity of the Shubarkol–Temirtau coke electrode sample (1:2) [$C_{p\;\pm \;}\bar{\delta }$, J/(g·K)].

T, K	$C_{p\pm }\bar{\delta }$
298	0.7545 ± 0.0151
323	0.9151 ± 0.0125
348	1.1184 ± 0.0152
373	1.2420 ± 0.0163
398	1.0410 ± 0.0212
423	1.1228 ± 0.0070
448	1.1994 ± 0.0229

**Table 7:** Dependence of electrical resistance (R), electrical capacity (C), and dielectric constant (ε) of the obtained electrode coke on temperature (T) at a frequency of 1 kHz.

T, K	C, nF	δ_C_	R, Oм	δ_R_	ε	lgε	lgR
1 kHz							
293	3684.2	3.02	3650	3.074	31,818,633	7.50	3.56
303	369.89	3.60	6032	3.288	3,194,559	6.50	3.78
313	230.26	4.78	4784	3.847	1,988,643	6.30	3.68
323	412.8	3.88	2969	3.578	3,565,152	6.55	3.47
333	636.58	3.84	1580	3.184	5,497,830	6.74	3.20
343	745.88	3.55	1370	2.304	6,441,801	6.81	3.14
353	862.67	3.17	1082	3.245	7,450,459	6.87	3.03
363	1000	3.50	896.4	2.084	8,636,511	6.94	2.95
373	1093.1	3.21	792.6	2.595	9,440,570	6.97	2.90
383	1002.9	3.54	967.5	4.946	8,661,557	6.94	2.99
393	1658.1	3.62	1236	4.731	14,320,199	7.16	3.09
403	10,790	3.76	715.5	4.750	93,187,952	7.97	2.85
413	11,253	3.11	768.5	3.179	97,186,657	7.99	2.89
423	34,036	3.82	598.4	3.959	293,952,283	8.47	2.78
433	56,061	3.49	502.7	6.220	484,171,435	8.68	2.70
443	82,216	3.61	321.3	3.234	710,059,376	8.85	2.51
453	99,999˂	-	237.6	4.812	863,642,449˂	8.94˂	2.38
463	99,999˂	-	193.9	3.198	863,642,449˂	8.94˂	2.29
473	99,999˂	-	131.2	2.934	863,642,449˂	8.94˂	2.12
483	99,999˂	-	102.3	2.499	863,642,449˂	8.94˂	2.01

**Table 8:** Dependence of electrical resistance (R), electrical capacity (C), and dielectric constant (ε) of the obtained electrode coke on temperature (T) at a frequency of 5 kHz.

T, K	C, nF	δ_C_	R, Oм	δ_R_	ε	lgε	lgR
5 kHz							
293	308.65	6.573	3828	4.562	2,665,659	6.43	3.58
303	46.172	5.781	6511	2.599	398,765	5.60	3.81
313	57.811	4.162	4566	4.042	499,285	5.70	3.66
323	91.109	0.476	1995	1.034	786,864	5.90	3.30
333	169.17	3.854	1714	9.181	1,461,039	6.16	3.23
343	187.13	4.632	1114	1.368	1,616,150	6.21	3.05
353	216.48	0.525	907.9	3.789	1,869,632	6.27	2.96
363	259.86	6.542	745.2	2.017	2,244,284	6.35	2.87
373	284.16	8.275	666.6	2.526	2,454,151	6.39	2.82
383	212.56	2.928	858.9	2.622	1,835,777	6.26	2.93
393	289.78	4.538	1145	4.389	2,502,688	6.40	3.06
403	1545.3	3.891	701.9	2.222	13,346,000	7.13	2.85
413	1510.6	4.679	774.5	3.412	13,046,313	7.12	2.89
423	5571.9	2.702	560.2	0.803	48,121,775	7.68	2.75
433	5677.7	1.646	490.6	7.313	49,035,518	7.69	2.69
443	11,863	2.969	326.6	1.257	102,454,928	8.01	2.51
453	30,077	1.535	226.6	3.521	259,760,337	8.41	2.36
463	48,569	4.794	187.8	2.340	419,466,696	8.62	2.27
473	72,034	2.520	129.4	5.026	622,122,423	8.79	2.11
483	99,999	0.695	100.7	4.975	863,642,449˂	8.94˂	2.00

**Table 9:** Dependence of electrical resistance (R), electrical capacity (C), and dielectric constant (ε) of the obtained electrode coke on temperature (T) at a frequency of 10 kHz.

T, K	C, nF	δ_C_	R, Oм	δ_R_	ε	lgε	lgR
10 kHz							
293	87.483	2.707	4054	2.874	755,548	5.88	3.61
303	16.326	6.811	5628	2.715	140,999	5.15	3.75
313	23.399	2.964	4018	1.922	202,086	5.31	3.60
323	48.044	2.885	2530	4.798	414,933	5.62	3.40
333	97.177	4.134	1422	1.657	839,270	5.92	3.15
343	111.81	3.452	1027	3.685	965,648	5.98	3.01
353	118.56	5.179	836	0.436	1,023,945	6.01	2.92
363	146.11	6.405	686.6	4.310	1,261,881	6.10	2.84
373	154.16	1.445	620.6	1.134	1,331,405	6.12	2.79
383	105.72	2.075	836.8	4.077	913,052	5.96	2.92
393	133.02	6.096	1104	3.965	1,148,829	6.06	3.04
403	630.9	1.367	690.2	5.755	5,448,775	6.74	2.84
413	611.17	1.489	750.4	4.312	5,278,376	6.72	2.88
423	1734.7	5.784	530.9	4.371	14,981,755	7.18	2.73
433	2348	5.073	462.5	3.712	20,278,527	7.31	2.67
443	6088.7	3.004	310.8	3.484	52,585,124	7.72	2.49
453	9926.8	5.372	208.1	7.654	85,732,916	7.93	2.32
463	20,357	3.305	183.6	2.579	175,813,451	8.25	2.26
473	34,701	2.419	123.1	2.813	299,695,563	8.48	2.09
483	55,279	2.797	98.6	2.719	477,417,683	8.68	1.99

**Table 10:** Yield of resin fractions of JSC “Shubarkol Komir”.

№	Fraction, with T_boiling_, °C	Mass, g	Output, %
1	Up to 150	17.00	18.47
2	170–190	23.00	25.00
3	220–250	4.00	4.35
4	Solid residue (pitch)	48.00	52.17

## Data Availability

Data are contained within the article.
